# Why We Need Urban Health Equity Indicators: Integrating Science, Policy, and Community

**DOI:** 10.1371/journal.pmed.1001285

**Published:** 2012-08-14

**Authors:** Jason Corburn, Alison K. Cohen

**Affiliations:** 1 University of California Berkeley, Department of City and Regional Planning & School of Public Health, Berkeley, California, United States of America; 2 University of California Berkeley, School of Public Health, Division of Epidemiology, Berkeley, California, United States of America

## Abstract

Jason Coburn and Alison Cohen discuss the need for urban health equity indicators, which can capture the social determinants of health, track policy decisions, and promote greater urban health equity.

**Summary Points**

- As the urban population of the planet increases and puts new stressors on infrastructure and institutions and exacerbates economic and social inequalities, public health and other disciplines must find new ways to address urban health equity.
- Urban indicator processes focused on health equity can promote new modes of healthy urban governance, where the formal functions of government combine with science and social movements to define a healthy community and direct policy action.
- An inter-related set of urban health equity indicators that capture the social determinants of health, including community assets, and track policy decisions, can help inform efforts to promote greater urban health equity.
- Adaptive management, a strategy used globally by scientists, policy makers, and civil society groups to manage complex ecological resources, is a potential model for developing and implementing urban health equity indicators.
- Urban health equity indicators are lacking and needed within cities of both the global north and south, but universal sets of indicators may be less useful than context-specific measures accountable to local needs.

## Toward Healthier and More Equitable Cities

As the world urbanizes, global health challenges are increasingly concentrated in cities. Currently, over 80% of the population in Latin America already lives in cities. The African urban population is projected to double in the next decade and China has urbanized in thirty years at a rate it took Europe and North America a century [1]. Rapidly growing new cities and increasingly segregated older cities in the global north and south are contributing to health inequities. Urban planning and policies can influence population health by supporting or stymieing opportunities for employment, housing security, political participation, education, protection from environmental risks, access to primary health care, and a host of other social and physical determinants of well-being [2]. As the World Health Organization (WHO) and UN-HABITAT acknowledged in the 2010 report entitled “Hidden Cities: Unmasking and Overcoming Health Inequities in Urban Settings”, where in a city you live and how that city is governed can determine whether or not one benefits from city living [3].

Measuring the forces that contribute to urban health is one challenge for promoting more healthy and equitable cities. Burden of disease estimates have tended to focus on the whole world or specific geographic regions [4],[5]. These data can mask intra-city differences and global data may not be relevant to inform national or municipal policy making. Public health has developed metrics for single pathogenic exposures or risk factors, but these measures often ignore both community assets that promote health equity and the cumulative impacts on health from exposure to multiple urban environmental, economic, and social stressors [6],[7]. Recognizing these population health challenges, the United Nations (UN) Commission on Social Determinants of Health (2008) called for “health equity to become a marker of good government performance” ([8], p. 11) and for the UN to “adopt health equity as a core global development goal and use a social determinants of health indicators framework to monitor progress” ([8], p. 19). More recently, the 2011 World Social Determinants of Health Conference and the Pan-American Health Organization's Urban Health Strategy called for the development of new urban health equity indicators that track the drivers of health inequities across place and time, particularly within a city neighborhood [9] (Box S1). In this paper, we briefly outline an approach for promoting greater urban health equity through the drafting and monitoring of indicators. We draw examples from the cities of Richmond, California, and Nairobi, Kenya. More specifically, we argue that participatory indicator processes hold the potential to shape new healthy and equitable urban governance by:

1. integrating science with democratic decision making;
2. tracking policy decisions that shape the distribution of health outcomes; and
3. including protocols for ongoing monitoring and adjusting of measures over time.

## Indicators for Health Promoting Policy Change

Ongoing measurement and evaluation is one critical aspect of moving toward more healthy and equitable cities because what we measure often matters for whether and how we act. Yet, the danger of indicator efforts is that they portray a too simplified picture of a complex reality and policy solutions may suffer the same defects. For example, indicators of single chemical exposures cannot produce policy-relevant knowledge about the environmental health consequences of multiple exposures. In a similar way, cross-sectional measures of single built and social environmental features of urban neighborhoods tend to ignore the cascading and relational effects of inequalities in urban areas. For example, in our own work in the slums of Nairobi, we have found that typical indicators that only measure population access to a toilet can misconstrue whether an ablution block is hygienic or safe. In Nairobi's slums, accessing a toilet may be controlled by a local cartel that might extort a high price for users, disproportionately impacting family income, while at the same time acting as a location for rape and sexual violence against women, particularly at night, when the toilet has no lighting or security, which in-turn might contribute to the spread of sexually transmitted diseases. Capturing the relationships between a physical or economic measure, the political decisions that shape the distribution of community resources, and how urban residents currently navigate urban inequities to stay healthy is central to our understanding of effective and meaningful urban health equity indictors.

The WHO defines an indicator as a variable with characteristics of quality, quantity, and time used to measure, directly or indirectly, changes in health and health-related situations [10]. In this paper, we use the term indicator to represent a construct often consisting of more than one measure, with a metric being the actual quantitative or qualitative data that is used to populate the indicator [11]. Our review of the vast health equity indicator literature suggests that indicators should do more than capture health outcomes, but also the determinants of health that often drive outcomes, including institutional practices and policy decisions made outside the health care and medical sectors. In addition, effective urban health equity indicators ought to highlight associations between determinants and health impacts, use data that are verifiable and easily accessible, and be shared in a clear and compelling way to a range of interested stakeholders [11],[12],[13],[14] (Table 1 and S1).

**Table 1:** Comparison of conventional and our approach to urban health equity indicators.

Characteristic	Conventional Health Status Indicator	Urban Health Equity Indicators
Time	Cross-sectional	Longitudinal: tracks progress over time
Orientation	Deficit-based	Asset-driven: strikes balance between identifying problems & building upon strategies that are already working
Levels	Individual behavior & biologic	Focuses on individual & community characteristics plus local, national, and international policies
Populations & places	Static	Dynamic: acknowledges that populations change and that definitions of community will change
Accessibility	Expert-driven	Collaborative & participatory between professionals & community members
Policy relevance	Health sector	Explicitly linked to policy making institutions within and outside the health sector
Political power	Unclear	Emphasizes accountability and transparency in political process and distribution of state resources

## Indicators as Adaptive Urban Health Equity Governance

The complexity of cities and the variegated forces that contribute to (in)equity in urban neighborhoods demands that indicator development processes are similarly dynamic. The drafting, measuring, tracking, and reporting of indicators can be viewed not as a technical process for experts alone, but rather as an opportunity to develop new participatory science policy making, or what we call governance. Governance is not just government and the decisions of formal institutions, such as ministries of health, but also includes the norms, routines, and practices that help shape which issues get onto the health research and policy agenda, what evidence base is used to underwrite decisions, and which social actors are deemed expert enough to participate in these decisions [15]. In other words, governance processes can shape what issues are deemed important for promoting health equity and which institutions are responsible for action [16] (Box 1).

**Box 1. How Indicators Act as a Form of Healthy Urban Governance**

1. Identifying and framing what counts as a health policy issue.
2. Generating, or contributing to, the evidentiary standards that underwrite health equity issues.
3. Constituting some social actors as “experts”, by deciding who gets to participate in defining indicators.
4. Grappling with different knowledge claims as the weight and importance of indicators is debated.
5. Highlighting the importance of public accountability and transparency of data in the way indicators are reported and shared with various publics.

The UN-HABITAT Urban Indicators project recognizes the importance of governance measures for tracking urban equity. In order to measure governance, the UN project measures such things as the degree of decentralization in public decision making, voter participation, the number of participants in civil society organizations, and the public transparency and accountability of local government institutions [17]. Similarly, the World Health Organization's Urban Health Equity Assessment and Response Tool (HEART) also attempts to measure some aspects of governance related to health equity and includes indicators such as government spending on health and education, voter participation, percentage of population completing primary education, and the proportion of the population covered by health and other insurance [18]. These are important steps in acknowledging and capturing the role of politics and non–health care specific policy making in measuring and acting to promote greater health equity in cities.

We suggest that indicator processes themselves, not just the measures, can act as opportunities for crafting new healthy and equitable urban governance. While this is an emerging idea for city health management, ecologists and others have used an iterative governance process called adaptive management for decades to steward complex ecosystems, such as forests, wetlands, and fisheries [19]. Adaptive management acknowledges the failures of linear processes where narrow disciplinary scientists have aimed to develop complex models, predict long-term outcomes, and suggest one-time policy standards. Instead, adaptive management begins with an acknowledgement of the inherent complexity and uncertainty within systems, that this complexity demands an iterative, ongoing learning process among a range of expert stakeholders, and that policy interventions must be adjusted to reflect newly acquired knowledge [20]. Another difference between adaptive management and conventional science policy is that adaptive management does not postpone actions until definitive causality is known about a system, but rather emphasizes the importance of action in the face of uncertain science and couples decisions tightly to rigorous monitoring [21],[22].

The process of adaptive management is one where a broad group of stakeholders, from scientists to policy makers to users of a resource, work together to generate evidence, make decisions, monitor the progress of those decisions, and make ongoing adjustments to decisions as new information emerges from monitoring [20]. Gohlke and Portier call for greater capacity within public health institutions to adapt to new and emerging challenges, such as drug-resistant infections and climate change, and that the field and discipline is currently ill-suited for adaptive science-based research and practice [21]. Huang et al. also stress the importance of enhancing resources and training for the redesign of public health institutions to enhance the field's adaptive capacity [22]. Yet, few others in public health or urban planning have explored the potential of applying adaptive management to promote greater health equity in cities.

## Urban Health Inequities in North and South

Drawing from our collaborative work on healthy urban governance and the drafting of health equity indicators in Richmond, California, and the Mathare Valley informal settlement in Nairobi, Kenya, we offer some brief examples of what an urban health equity adaptive management strategy might entail.

In Richmond, California, located in the San Francisco Bay Area, one-third of residents live at or below 200% of the federal poverty line, over 60% of the population is African-American, Latino, or Asian-American, one in seven people are unemployed, residents live with elevated concentrations of industrial and mobile source air pollution, and it is one of the most violent cities, measured by per-capita homicide rates, in the United States [23]. In Richmond, African-Americans have the highest rate of infant mortality, low-birth weight babies, and asthma hospitalizations in Contra Costa County, and residents of the Iron Triangle neighborhood, one of the poorest in the city, die on average 13 years earlier than their wealthier white neighbors [23].

The Mathare Valley is a sprawling informal settlement in Nairobi, where over 82% of residents rent dirt floor, sheet metal–walled, one-room shacks and 88% lack access to clean and reliable drinking water or a private, hygienic toilet [24] (Figure S1). According to a 2011 household survey conducted in Mathare, over two-thirds of residents experienced routine violence in the last year, over half live on environmentally risky slopes and flood-prone areas, and households spend, on average, 76% of their monthly income on food [24]. Child mortality in Nairobi's slums is 151 per 1,000 live births, compared to 62 per 1,000 in all of Nairobi and 113 in rural Kenya [25].

Recognizing these health inequities and the multiple factors contributing to them, actions to improve the physical, social, and economic environments are occurring in both Richmond and Mathare. In Richmond, community groups and the city government drafted a Health and Wellness Element—or a development and policy blueprint—as part of the city's General Plan Update. Community-based organizations also led their own processes to collect data for and draft health equity indicators [26],[27].

In Mathare, community groups are working to reduce violence, engage youth in employment activities, and build toilets and schools [28],[29]. One coalition includes Muungano wa Wanavijiji, the federation of the urban poor in Kenya, Slum Dwellers International (SDI), the University of Nairobi, and the University of California, Berkeley, who together are organizing residents to plan for physical and social improvements that include new water and sanitary infrastructure, housing and land rights, and environmental and health care services [30],[31]. The government of Kenya and the World Bank recently launched a national policy initiative aimed at improving living conditions and well-being in slums, called the Kenya Informal Settlements Improvement Programme (KISIP) [32].

## Urban Health Equity Indicators in Practice

In Richmond, the indicator process emerged from ongoing community organizing and land use planning, and included community-based organizations and the city and county health department. Community priorities were highlighted through a process called “Measuring What Matters” where over ten different community-based organizations identified priority issues, chose indicators, collected and analyzed data, and published a comprehensive report that included quantitative and qualitative information [27]. At the same time, the city organized a participatory process to draft and implement the Health and Wellness Element, which included a set of goals and metrics aimed at promoting and monitoring progress on population health [33]. In order to track and monitor indicators on an ongoing basis, the Richmond Health Equity Partnership (http://richmondhealth.org) was established in 2012 and includes representatives from the city, county health department, school district, and a host of community-based organizations.

In Mathare, the nongovernmental organization Muungano Support Trust (MuST) has organized residents to survey themselves and document community assets and vulnerabilities in three waves starting in 2007 through 2012 [28],[31]. These data have been combined with spatial maps of community assets and hazards and used by MuST in community planning processes focused on specific projects, such as improving housing, water infrastructure, health care access, and community facilities [31]. In 2011, a comprehensive slum redevelopment plan focused on Mathare was drafted by residents, MuST, the University of Nairobi, and University of California, Berkeley, which includes indicators and a process for ongoing monitoring [31].

In both cases, we began by organizing community health priorities into three broad health equity categories: living conditions, economics and services, and political power and outcomes. Under each category, indicators were selected that constituted the elements of the category. For instance, under living conditions, housing, key utilities such as water, sanitation and food, the physical environment, community safety, and transportation were selected as representative indicator categories (Table 2).

**Table 2:** Examples of urban health equity indicators.

Equity Category	Indicators	Example Measures for Richmond, CA	Example Measures for Mathare Informal Settlement, Nairobi, Kenya
**Living conditions**	**Housing**	• Percentage of eligible residents receiving housing subsidies (i.e., Section 8)• Number of rehabilitated, formerly foreclosed/vacant housing properties	• Percentage residents in savings program for housing• Ratio of structure owners to tenants
	**Water, sanitation, & food**	• Ratio of eligible persons to number receiving food supports• Self-reports of food insecurity	• Self-reports of food insecurity• Percent of households with in-home water & toilet service• Number of new electricity connections installed by utility company
	**Environment**	• Percentage households reporting air pollution or noise-altered sleep, concentration, or work/school performance.	• Number of infrastructure projects launched to secure housing on steep slopes & in flood areas• Number of non-charcoal burning cook-stoves sold at subsidized cost
	**Safety**	• Perception of safety, especially at night• Percentage participating in community policing/cease-fire activities	• Self reports of safety & violence from women
	**Transportation**	• Public spending on bus and rail transport as ratio of highway spending	• Public spending on transport
**Economics and services**	**Primary health care**	• Percent of adults who did not seek medical care because of the cost• Number of new community health workers at clinics & other providers	• Percentage free clinics offering maternal and childhood care using in-home community health workers
	**Mental/substance care**	• Percentage county budget funding formerly incarcerated community members to receive counseling & care	• Percent of international health research budgets spent on mental health services/interventions
	**Education**	• Percentage subsidized enrollment in youth after school programs	• Percent families receiving free day care
	**Employment**	• Percent local employers offering living wage jobs, paid sick days, & health care/insurance	• Percent of local residents hired to work on government and internationally funded contracts in past year
	**Wealth access**	• Number of new business permits issued by the city• Amount of Community Reinvestment Act funds spent in city	• Ratio of slum dwellers' new bank accounts to all new accounts by local banks in past year
**Political power & outcomes**	**Community participation**	• Number of community members & local organization representatives elected and/or appointed to city and county boards & commissions	• Percentage of residents participating in community-based organization
	**Government responsiveness**	• Percentage of public works complaints responded to within 30 days or less• Percentage of public participation processes that are held at convenient times, and provide transportation & language translation	• Number of meetings held in community by Nairobi's city council and water & power company addressing ongoing infrastructure, housing, & health issues
	**Recognition of minority rights (women)**	• Percentage of residents reporting experiences of gender or ethnic discrimination in school, government relations, police interaction, and/or workplace	• Number of women given land rights/housing tenure by City Council
	**Health status**	• Self-rated health	• Self-rated health
	**Art/cultural expression**	• Per-capita funding for the arts	• Percentage of youth and adults participating in cultural programs

Importantly for public health practitioners, policy makers, and community residents, each indicator includes a health- and equity-based rationale that is referenced in the peer-reviewed literature. While a number of measures could populate each indicator, the participatory process selected one or two priority measures that were deemed representative of the larger equity issue being addressed and linked these to local or state policies that were understood by participants as potentially promoting greater health equity. The idea was to generate a set of measures that *when combined* could suggest whether or not the community was making progress toward greater health equity (Figure S2).

## Limits of City Health Equity Indicators

All indicator efforts are limited in that they make judgments about selecting and highlighting certain data over others. These value judgments do not make indicator efforts unscientific or invalid, but rather demand that the processes for selection and the denial of data be explicit and transparent and, as we have suggested here, open to interpretation and re-evaluation. Another limitation of indicator efforts is whether they inspire action by different actors, from within and outside the health sector. Traditional indicators that measure morbidity and mortality tend to either place responsibility for improving health on the medical and public health communities or on vaguely identified institutions such as the economy, education, or built environment. The result is an overemphasis on medical and public health solutions while failing to articulate the specific institutions and policies that might need to change to promote greater health equity. While our examples from Richmond and Nairobi are in the early stages, we are witnessing non–health care specific sectors and institutions, from urban planning to legal and housing rights to violence prevention, re-framing their work as contributing to urban health equity.

Indicator projects can also be limited by a lack of available data and the costs of obtaining locally specific information. Very few cities in the global north or south collect data on the social determinants of health at the neighborhood scale and those that do rarely keep these data in one publically accessible location. However, advances in mobile information technology and the use of hand-held devices with built-in sensors are creating new opportunities for tracking and reporting different types of urban health equity data. In Toronto, Canada, the health ministry has created the Toronto Central Local Health Integration Network (LHIN), which aims to bring together multiple community actors and government agencies to improve health for the urban poor and tracks progress using indicators of equity [34]. In Rio de Janeiro, Brazil, the Center for Health Promotion is a network of over 150 civil society organizations working to promote health equity and, among other tasks, gathers data on the social determinants of health equity in Rio's favelas [35]. In Belo Horizonte, Brazil, an urban health observatory conducts participatory research and maintains data on population- and place-based health equity issues [36]. The Indian nongovernmental organization Urban Health Resource Centre (http://www.uhrc.in) works with the urban poor to improve health equity and helped shape India's National Urban Health Mission, which will document many determinants of health in cites [37]. In San Francisco, California, the public health department maintains a health equity–oriented publically available database called the Healthy Development Measurement Tool [38].

## Conclusions

As urban health is increasingly recognized as a global health priority, new indicators accompanied by monitoring processes that can adapt and improve over time will be necessary to promote greater health equity. We have suggested here that indicator processes might be one important strategy to encourage new models of urban health governance in both the global north and south. Like any concept, more research and evaluation is necessary to understand the barriers and opportunities for turning our conceptual ideas into practice. Yet, lessons from other fields and emerging experiments around the world suggest that indicator processes can integrate science, policy, and community to promote greater urban health equity.

## Supporting Information

Box S1 **The importance of urban health equity indicators.**
(DOC)

Figure S1 **Map of Nairobi and Mathare informal settlement.**
(TIF)

Figure S2 **Relational “diamond” of urban health equity indicators.**
(TIF)

Table S1 **Characteristics of successful urban health equity indicators.**
(DOC)

## Abbreviations

MuST: Muungano Support Trust
UN: United Nations
WHO: World Health Organization
