# Supplementary material for: Why We Need Urban Health Equity Indicators: Integrating Science, Policy, and Community
Source: PLoS Med. 2012 Aug 14;9(8):e1001285. doi: 10.1371/journal.pmed.1001285 (PMC3419162; doi:10.1371/journal.pmed.1001285)
Supplement: Box S1 — The importance of urban health equity indicators. (DOC) [file pmed.1001285.s001.doc]

Box: The Importance of Urban Health Equity Indicators

The 2010 WHO and UN-HABITAT report, *Hidden Cities*, notes:

Understanding urban health begins with knowing which city dwellers are affected by which health issues, and why. To achieve this understanding, available information must be disaggregated according to defining characteristics of city dwellers, such as their socioeconomic status or place of residence…Disaggregated data invariably reveal urban health inequities, which are defined as health inequalities that are systemic, socially produced (and therefore modifiable) and unfair. Health inequities are the result of the circumstances in which people grow, live, work and age, and the health systems they can access, which in turn are shaped by broader political, social and economic forces. They are not distributed randomly, but rather show a consistent pattern across the population, often by socioeconomic status or geographic location. No city – large or small, rich or poor, east or west, north or south – has been shown immune to the problem of health inequity (pxii).
