# Supplementary figures and images for: Why We Need Urban Health Equity Indicators: Integrating Science, Policy, and Community
Source: PLoS Med. 2012 Aug 14;9(8):e1001285. doi: 10.1371/journal.pmed.1001285 (PMC3419162; doi:10.1371/journal.pmed.1001285)

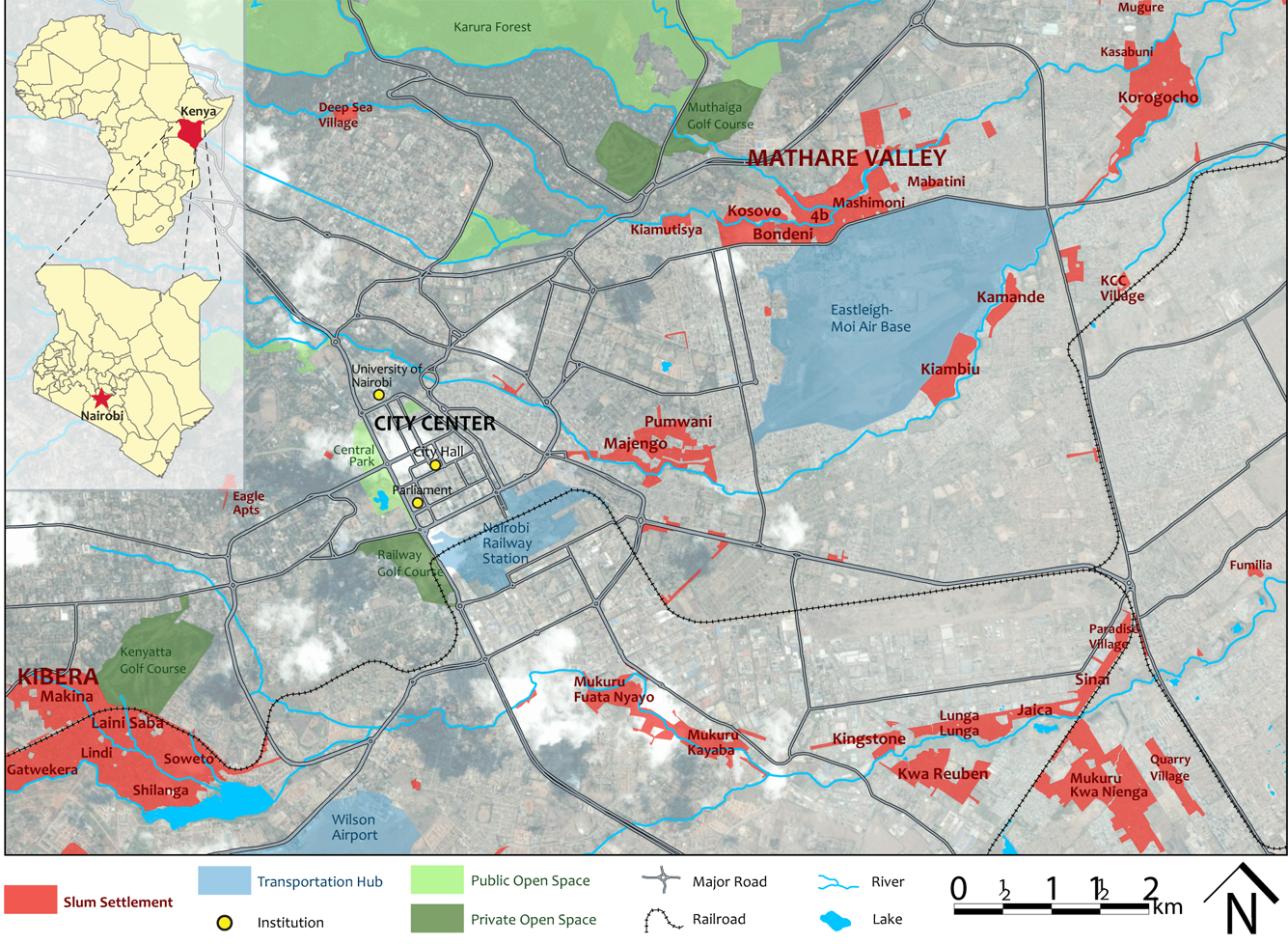

Supplement: Figure S1 — Map of Nairobi and Mathare informal settlement. (TIF) [file pmed.1001285.s002.tif]

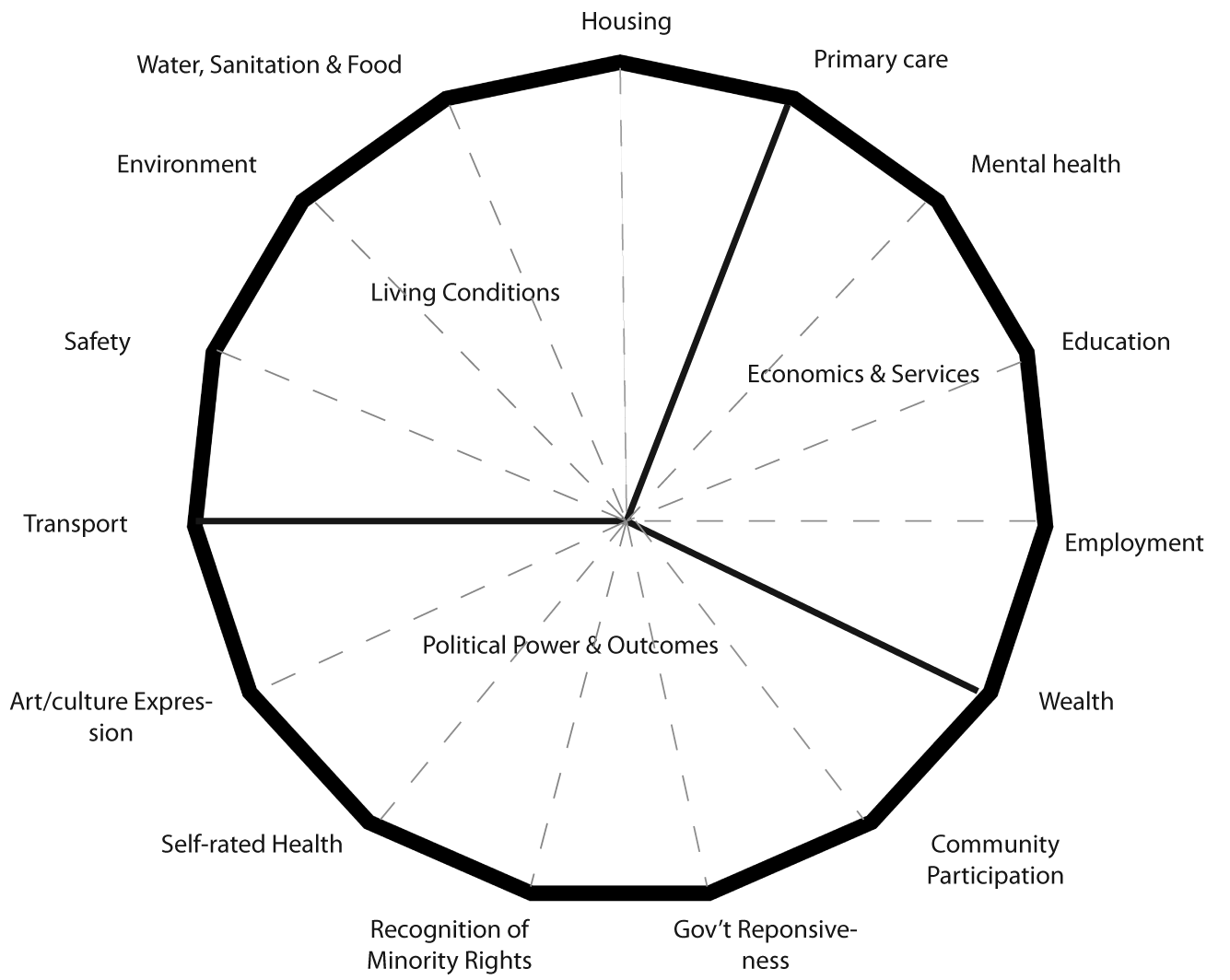

Supplement: Figure S2 — Relational “diamond” of urban health equity indicators. (TIF) [file pmed.1001285.s003.tif]
