# Supplementary material for: Why We Need Urban Health Equity Indicators: Integrating Science, Policy, and Community
Source: PLoS Med. 2012 Aug 14;9(8):e1001285. doi: 10.1371/journal.pmed.1001285 (PMC3419162; doi:10.1371/journal.pmed.1001285)
Supplement: Table S1 — Characteristics of successful urban health equity indicators. (DOC) [file pmed.1001285.s004.doc]

Table: Characteristics of Successful Urban Health Equity indicators

| **Characteristic** | **Description** |
| --- | --- |
| **Simplicity** | Easy to understand by a range of people and not requiring too much expert knowledge; tap into local knowledge and community expertise. |
| **Relevance** | They illustrate something about the system that residents and/or decision-makers need to know, and are collected frequently enough to influence action. They may also inspire people to see circumstances in new ways, including by disaggregating information by population groups, places, and/or presenting trends over time. |
| **Reliability** | Measures both assets and liabilities; public trust behind the information/methods of data collection; easily verifiable through available data. |
| **Accessibility** | The information is already available or regularly gathered and made publically available. |
| **Responsibility** | Identifies specific institutions, policies or processes that historically and currently contribute to inequities and those that work to promote greater equity. |
| **Relational & Adaptable** | Together, a set of indicators tell a compelling story about health equity; focus on relationships among measures, not just individual metrics. |
| **Reporting** | Develops reporting strategy from the beginning, identifies intended audiences, utilizes multiple forms of media/technology, and timed to inform various research and political decision-making processes. |
